# Update of a prognostic survival model in head and neck squamous cell carcinoma patients treated with immune checkpoint inhibitors using an expansion cohort

**DOI:** 10.1186/s12885-022-09809-5

**Published:** 2022-07-14

**Authors:** Majd Issa, Brett G. Klamer, Nikol Mladkova, Georgios I. Laliotis, Vidhya Karivedu, Priyanka Bhateja, Chase Byington, Khaled Dibs, Xueliang Pan, Arnab Chakravarti, John Grecula, Sachin R. Jhawar, Darrion Mitchell, Sujith Baliga, Matthew Old, Ricardo L. Carrau, James W. Rocco, Dukagjin M. Blakaj, Marcelo Bonomi

**Affiliations:** 1 Department of Internal Medicine, Division of Medical Oncology, The Ohio State University Wexner Medical Center, Columbus, OH 43210 USA; 2 Center for Biostatistics, Department of Biomedical Informatics, The Ohio State University, Columbus, OH 43210 USA; 3 Division of Radiation Oncology, The Ohio State University Wexner Medical Center, Columbus, OH 43210 USA; 4 Sidney Kimmel Comprehensive Cancer Center and Department of Oncology, Johns Hopkins University, Baltimore, MD 21231 USA; 5 Department of Otolaryngology - Head and Neck Surgery, The Ohio State University Wexner Medical Center, Columbus, OH 43210 USA

**Keywords:** Head and neck cancer, Head and neck squamous cell carcinoma, Immunotherpay, Immune checkpoint inhibitors, Survival in head and neck cancer

## Abstract

**Background:**

Immune checkpoint inhibitors (ICI) treatment in recurrent/metastatic (R/M) head and neck squamous cell carcinoma (HNSCC) offers new therapeutic venues. We have previously developed a predictive survival model in this patient population based on clinical parameters, and the purpose of this study was to expand the study cohort and internally validate the model.

**Methods:**

A single institutional retrospective analysis of R/M HNSCC patients treated with ICI. Clinical parameters collected included p-16 status, hemoglobin (Hb), albumin (Alb), lactate dehydrogenase (LDH), neutrophil, lymphocyte and platelet counts. Cox proportional hazard regression was used to assess the impact of patient characteristics and clinical variables on survival. A nomogram was created using the *rms* package to generate individualized survival prediction.

**Results:**

201 patients were included, 47 females (23%), 154 males (77%). Median age was 61 years (IQR: 55-68). P-16 negative (66%). Median OS was 12 months (95% CI: 9.4, 14.9). Updated OS model included age, sex, absolute neutrophil count, absolute lymphocyte count, albumin, hemoglobin, LDH, and p-16 status. We stratified patients into three risk groups based on this model at the 0.33 and 0.66 quantiles. Median OS in the optimal risk group reached 23.7 months (CI: 18.5, NR), 13.8 months (CI: 11.1, 20.3) in the average risk group, and 2.3 months (CI: 1.7, 4.4) in the high-risk group. Following internal validation, the discriminatory power of the model reached a c-index of 0.72 and calibration slope of 0.79.

**Conclusions:**

Our updated nomogram could assist in the precise selection of patients for which ICI could be beneficial and cost-effective.

## Simple summary

The development and progression of head and neck squamous cell carcinoma (HNSCC) have been associated with local and systemic immunosuppressive effects. Immune checkpoint inhibitors (ICI) are currently approved as first and second line treatment for recurrent metastatic (R/M) HNSCC. We have previously developed a predictive survival model in this patient population based on clinical parameters. The purpose of this study was to expand the cohort and internally validate the model. The updated overall survival (OS) model includes age, neutrophil and lymphocyte counts, albumin, hemoglobin, LDH, and p-16 tumor status. We stratified patients into three risk groups based on this model. Median OS in the optimal risk group reached 23.7 months (CI: 18.5, NR), 13.8 months (CI: 11.1, 20.3) in the average risk group, and 2.3 months (CI: 1.7, 4.4) in the high-risk group.

## Background

The development and progression of HNSCC have been associated with local and systemic immunosuppressive effects, highlighting the strong potential of immunotherapy to improve clinical outcomes [1, 2]. Anti-programmed cell death protein 1 (a-PD1) antibodies Pembrolizumab and Nivolumab represent the first immunotherapeutic agents associated with survival benefits in R/M HNSCC [3–6]. Recently, Pembrolizumab received US regulatory approval as first line treatment for R/M HNSCC [7], while both Pembrolizumab and Nivolumab are currently approved as second line treatment in R/M HNSCC following progression after platinum-based therapy [5, 6].

Despite the promise and the clinical potential of ICI in R/M HNSCC, only 13 to 20% of patients will achieve a clinical response and the majority will progress within the first 2 years of starting ICI [5–7]. Additionally, a segment of patients will experience rapid progression and poor outcome following treatment with single agent a-PD1 [8–10]. This highlights the need for a reliable tool to identify potential candidates for treatment with ICI.

Several markers capable of predicting ICI response have been previously studied extensively including PD-L1 expression, tumor mutational burden, the presence of neo-antigens in tumor microenvironment (TME) and the expression of IFN-γ signature [11, 12]. Yet the assessment of these markers requires additional resources, substantial laboratory expertise and costly equipment, and their prognostic value in terms of overall survival (OS) remains uncertain.

The association between the inflammatory response, regulation, cancer development, progression, and immunotherapy response has been established previously. Persistent inflammation leads to tissue damage, and conversely, tissue regeneration. As part of the process, a plethora of signaling substances are released by inflammatory cells, leading to multiple systemic effects including genomic alterations and tumor progression [13]. Recent data uncovered two distinct microenvironment subtypes based on their mixtures of stromal elements. These are characterized by markers of an activated or exhausted immune response [14]. These findings could provide guidance into the personalization of treatment approaches for HNSCC. We have previously developed a prognostic multivariable survival model using standard and routinely accessible clinical inflammatory markers, including neutrophil and lymphocyte counts, Alb, Hb and LDH values along with p-16 tumor status [15]. Here, we attempt to internally validate the power of our predictive model by expanding our analysis to a larger cohort of 201 patients.

## Methods

This is an Institutional Review Board approved retrospective study of a single institution cohort of HNSCC patients who received at least one dose of a-PD1 antibody for R/M HNSCC as a first, second line treatment (and beyond), between January 15th 2016 and April 9th 2020. Inclusion criteria were age greater than 18 years and a pathologically confirmed HNSCC subsites of oropharynx, oral cavity, larynx, hypopharynx, nasopharynx, and paranasal sinuses, or a p-16 tumor positive HNSCC of unknown primary. Exclusion criteria included incomplete treatment records, unclear tumor histology, patients who received concurrent ICI with chemotherapy and those who were treated with ICI in the setting of a clinical trial. We collected the following variables: age at the time of starting ICI, sex, p-16 tumor status, AJCC 7 tumor stage at diagnosis, previous lines of therapy, subsequent chemotherapy, sites of progression, immunotherapy drug, baseline Alb, Hb, LDH, absolute neutrophil count (Neu), absolute lymphocyte count (Lymph) and platelet count, as recorded in the chart at the time of ICI initiation. P-16 status (positive or negative) was tested for each oropharynx tumor site, but was not tested for tumors of other locations and set as negative for these cases. Primary clinical outcome was OS, secondary endpoint was progression free survival (PFS). OS was defined as the time from ICI initiation and death from any cause. PFS was defined as the time from ICI initiation and disease progression or death from any cause, censored at the last follow-up.

### Statistical analysis

Statistical analysis was conducted using *R* (version 4.1.0) [16], with the *survival* [17] and *rms* [18] packages. Cox proportional hazard regression models were used to assess the effects of patient characteristics and other selected variables on OS and PFS [19]. Non-linear relationships with the log-hazard were assessed using natural cubic splines and kept if significant using Wald’s test. We started with 4 knot locations at the 0.05, 0.35, 0.65, and 0.95 quantiles and verified if a larger number of knots kept the same shape of relationship. We allowed removal of clinical variables which were not significant in the multivariable model, demonstrated little importance for prediction (as seen in the nomogram contribution), and would improve validation characteristics. Hazard ratio estimates of numeric variables are based on standardized data values. A nomogram was generated using the *rms* package to predict the overall survival of this patient population. The prediction accuracy was evaluated using Nagelkerke’s R^2^ statistic as an overall measure of model fit. This statistic is a measure of explained variation in the outcome where 0 indicates a completely uninformative model and 1 indicates perfect model fit. The c-index was used as a measure of discriminative ability of the model. A c-index of 0.5 suggests no discriminating ability, and 1 suggests perfect discrimination. Model calibration was assessed graphically using calibration plots containing smoothed estimates of the predicted survival probability and the corresponding observed Kaplan-Meier estimate at 6 months, 1-, and 2-year time points. Internal validation of the model performance characteristics was assessed with 1000 bootstrap replications with replacement. This method allows measurement of the optimism (bias of overfitting) of the original model by providing correction factors for the model performance statistics R,^2^ c-index, and calibration. We sought to produce a model in which the optimism corrected c-index was 0.7 or greater. As a final method of model validation, we calculated the nomogram total points for each patient, separated the patients into three groups using the 0.33 and 0.66 quantiles as cut-off points, and used Kaplan-Meier survival curves to evaluate survival outcomes of the pre-defined groups.

## Results

### Patients characteristics

**Fig. 1:**
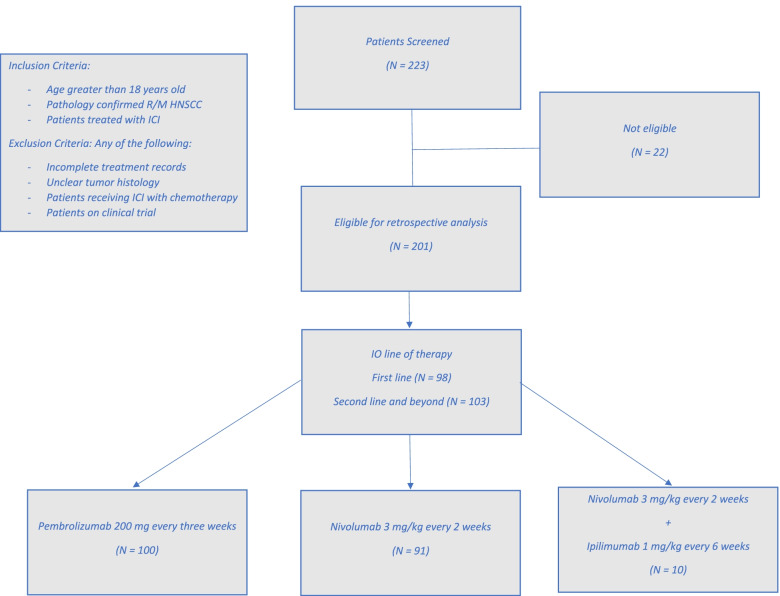
Study design

We analyzed data for a total of 223 patients that initiated treatment with ICI between January 15th 2016 and April 9th 2020, as first, second or further line of therapy. 201 patients met our inclusion criteria **(**Table 1**)**. There were 47 females (23%) and 154 males (77%), with a median age of 61 (IQR: 55-68). Sixty-nine patients (34%) had a p-16 positive tumor. A total of 84 (42%) tumors originated in the oropharynx, followed by 45 (22%) oral cavity, 26 (13%) larynx, and 46 (23%) malignancies originating from other sites. One hundred (50%) patients received pembrolizumab, 91 (45%) nivolumab, and 10 (5%) received a combination of nivolumab with ipilimumab. Ninety-eight (49%) patients received ICI as their first line of therapy, while 102 (51%) as a second line and beyond **(**Fig. 1**)**.

**Table 1:** Patients’ characteristics

Characteristic	N (%)
Sex	
Male	*154 (77%)*
Female	*47 (23%)*
Age	*61 (55-68)*
Tumor site	
Oropharynx	*84 (42%)*
Oral Cavity	*45 (22%)*
Larynx	*26 (13%)*
Other	*46 (23%)*
P-16 status	
Negative	*132 (66%)*
Positive	*69 (34%)*
IO line of therapy	
1	*98 (49%)*
2 and beyond	*103 (51%)*
Immunotherapy drug	
Pembrolizumab	*100 (50%)*
Nivolumab	*91 (45%)*
Nivolumab + Ipilimumab	*10 (5.0%)*
ECOG	
0	*36 (18%)*
1	*96 (48%)*
2 and 3	*69 (34%)*

Statistics presented: n (%)

Complete blood count with differential, comprehensive metabolic panel, and LDH level were obtained on the day of starting ICI treatment prior to drug administration. P-16 tumor status was assessed from the original biopsy. Our analysis revealed that patients had median Neu: 4.58 K/uL (IQR: 3.43-6.47), median Lymph: 0.69 K/uL (IQR: 0.47-1.08), Hb normal/low 101/100 (50%/50%), with a Hb value of 12.0 g/dL as a cut off, Alb: normal/low 156/45 (78%/22%) with an Alb level of 3.5 g/L as a cut off, LDH: normal/high 124/77 (62%/38%) with an LDH value of 190 U/L as a cut off.

### Main results

**Fig. 2:**
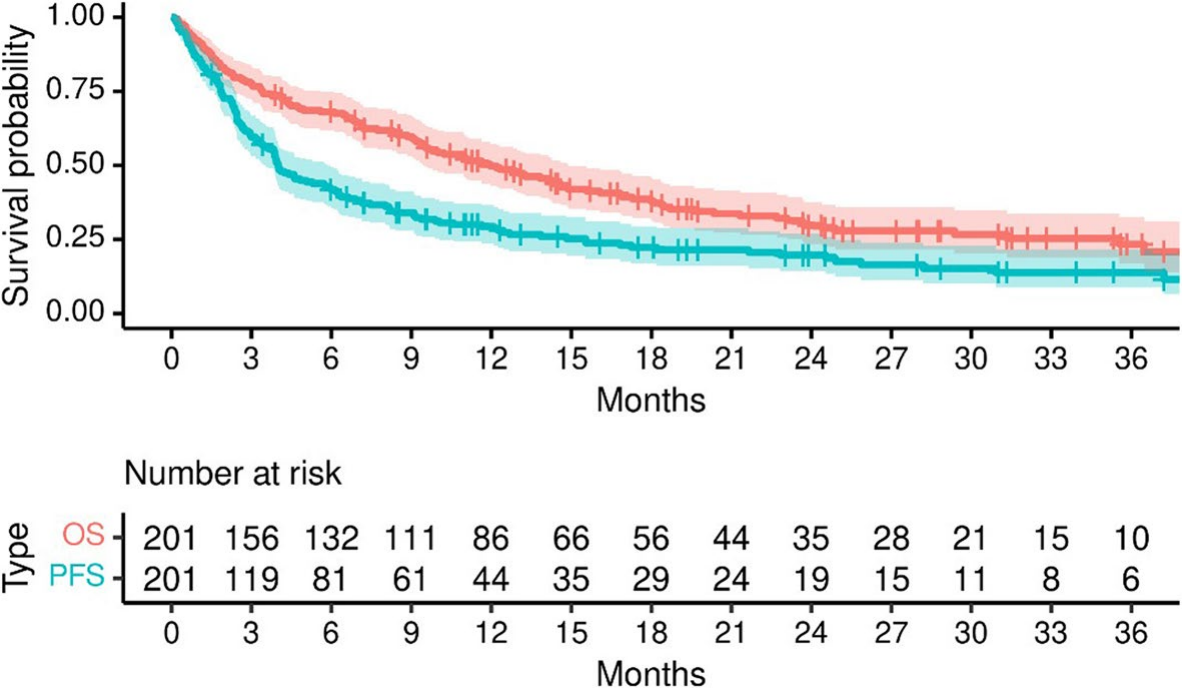
Overall survival (OS) and progression-free survival (PFS) for 201 patients treated with ICI as first, or second line and beyond

**Fig. 3:**
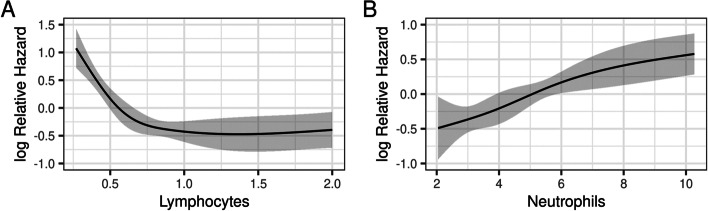
Impact of Lymphocyte (3A) and Neutrophil (3B) counts on Overall Survival

One hundred and twenty-four (62%) patients had progressed while treated with ICI, with 64 (32%) of them receiving subsequent chemotherapy. The most common type of recurrence on ICI was observed in distant sites in 83 (53%) patients. At the time of this analysis, 135 (67%) patients had expired. The Kaplan-Meier estimate of median OS was 12 months (95% CI: 9.4, 14.9) and median PFS was 4 months (95% CI: 3.5, 5.9) **(**Fig. 2**)**. We observed no significant difference in OS between patients on 1st line of therapy compared to 2nd line or greater (log rank: *p* = 0.5). The updated cohort provided 80 new patients for temporal validation. Using our original model to predict outcomes for this cohort [15], we found a slightly lower measure of discrimination and similar calibration slope compared to the original internally validated estimates (original internally validated c-index = 0.70 vs. temporal validated c-index = 0.68 and original internally validated calibration slope = 0.68 vs. temporal validated calibration slope = 0.69). In accordance with our previous approach [15], we explored Cox proportional hazard regression models using age, Neu, Lymph, platelet count, Alb, Hb, LDH and p-16 tumor status as predictor variables. Platelet count was removed from the updated model to improve internal validation characteristics and allow for non-linear relationship of Lymph with the log relative hazard (*p* < 0.001, Fig. 3**)**.

**Fig. 4:**
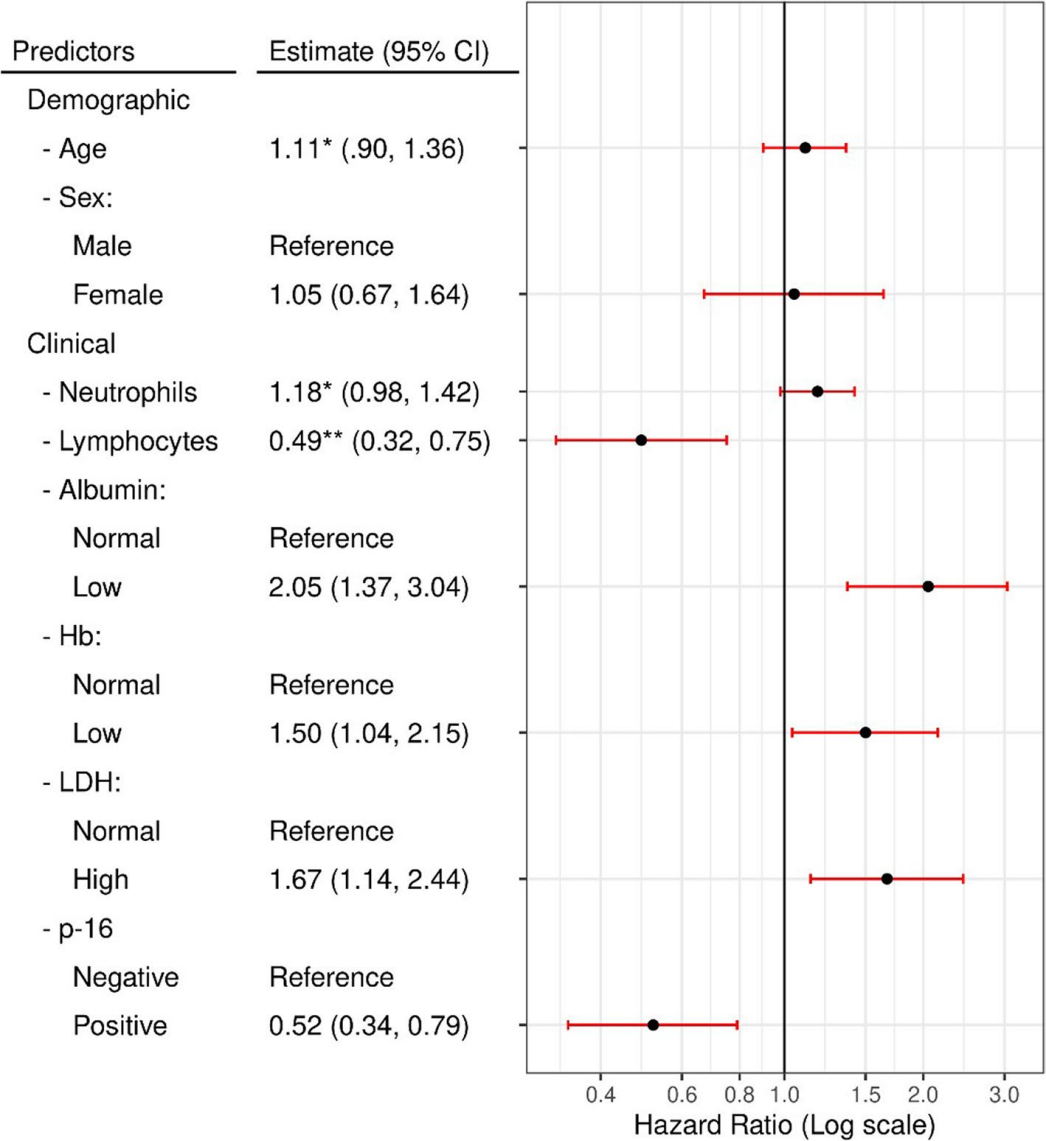
Results of multivariable cox hazards regression model. CI: confidence interval; *: coefficient based on standardized data; **: coefficient for the 3rd quartile compared to 1st quartile of the standardized data

Consistent with our previous findings, our updated analysis shows that Neu: [HR: 1.18; 95% CI: 0.98, 1.42; *p* = 0.08], Lymph: [3rd vs. 1st quartile; HR: 0.49; 95% CI: 0.32, 0.75], Alb (low) [HR: 2.05; 95% CI: 1.37, 3.04; p < 0.001], Hb (low) [HR: 1.50; 95% CI: 1.04, 2.15; *p* = 0.029], LDH (high) [HR: 1.67; 95% CI: 1.14, 2.44; *p* = 0.009] and p-16 tumor status (positive) [HR: 0.52; 95% CI: 0.34, 0.79; *p* = 0.002] were predictors of OS **(**Fig. 4**).**

**Fig. 5:**
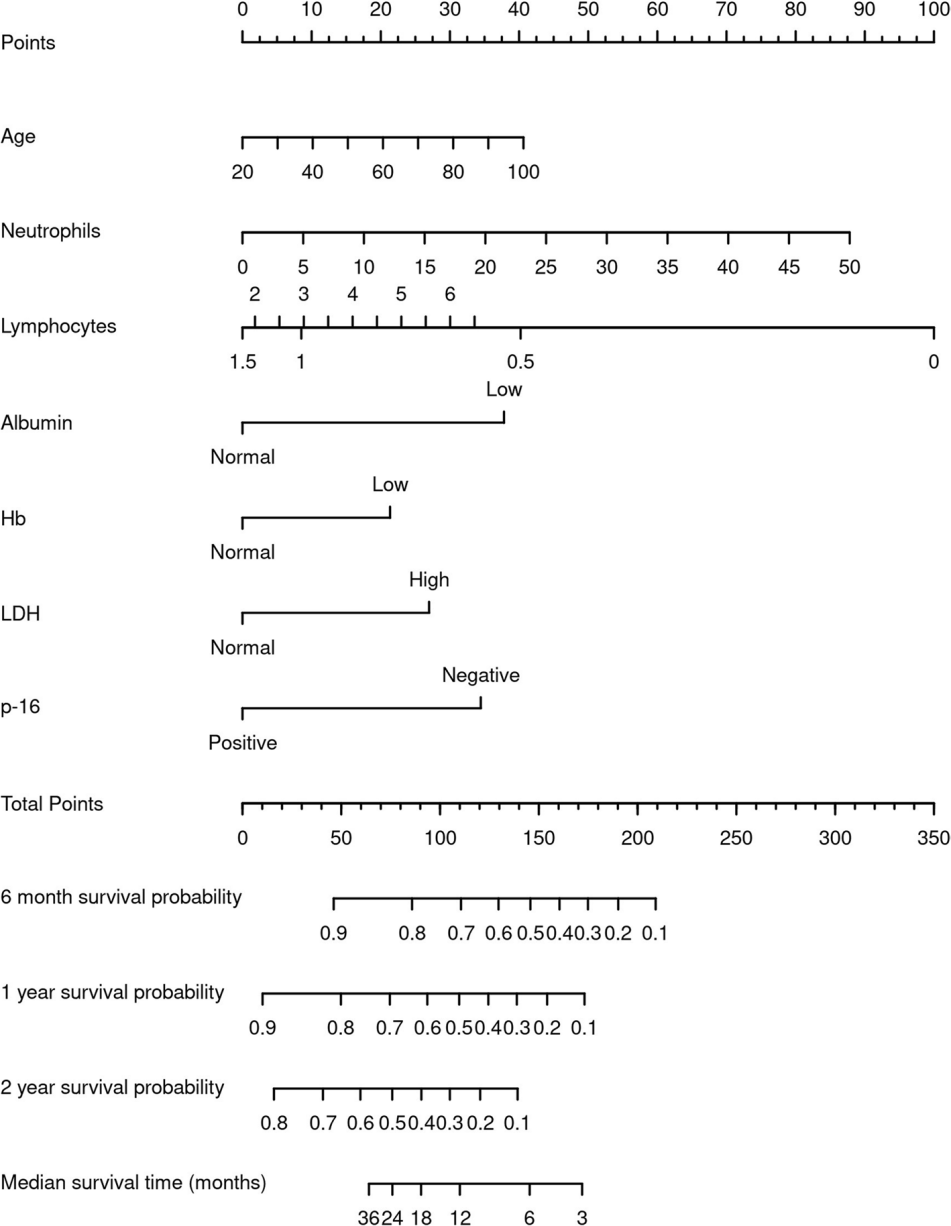
Nomogram of overall survival in our cohort

The expanded model was used to create a nomogram-based prognostic score **(**Fig. 5**)**. We believe the nomogram is a reliable and accessible method for clinicians to calculate survival predictions based on a patient’s pre-treatment characteristics. In order to obtain a patient’s prognostic score, a vertical line for the observed value of each prognostic variable to the “Points” line should be drawn. Subsequently, the values on the “Points” line should be summed to obtain the total points. Finally, a drawn vertical line from the “Total Points” line down to the survival lines corresponding to 6 months, 1 year, and 2 year timelines, will provide the predicted survival probability or median survival time.

**Fig. 6:**
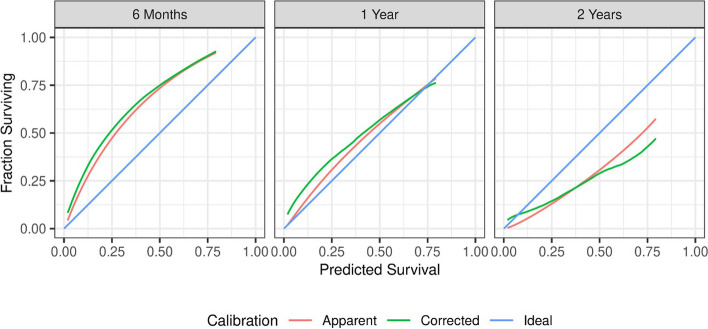
Internally validated measures of model calibration. Plots show higher than expected survival probability at 6 months, slightly higher than expected survival at 1 year, and lower than expected survival probability at 2 years. Apparent: model based fit; Corrected: bootstrap corrected model fit; Ideal: Ideal fit

To evaluate the performance of the model, bootstrapping was performed for internal model validation. The optimism adjusted measure of discrimination was adequate, with a c-index of 0.72. Apparent and optimism adjusted estimates of calibration are shown in Fig. 6. Predicted survival corresponded well to observed survival at the 1 year time point. At 6 months, predicted survival was lower than observed, while at 2 years, predicted survival was higher than observed. The optimism adjusted calibration slope at 1-year survival time and R^2^ were 0.79 and 0.25, respectively, both reflecting improvements compared to the originally developed model.

**Fig. 7:**
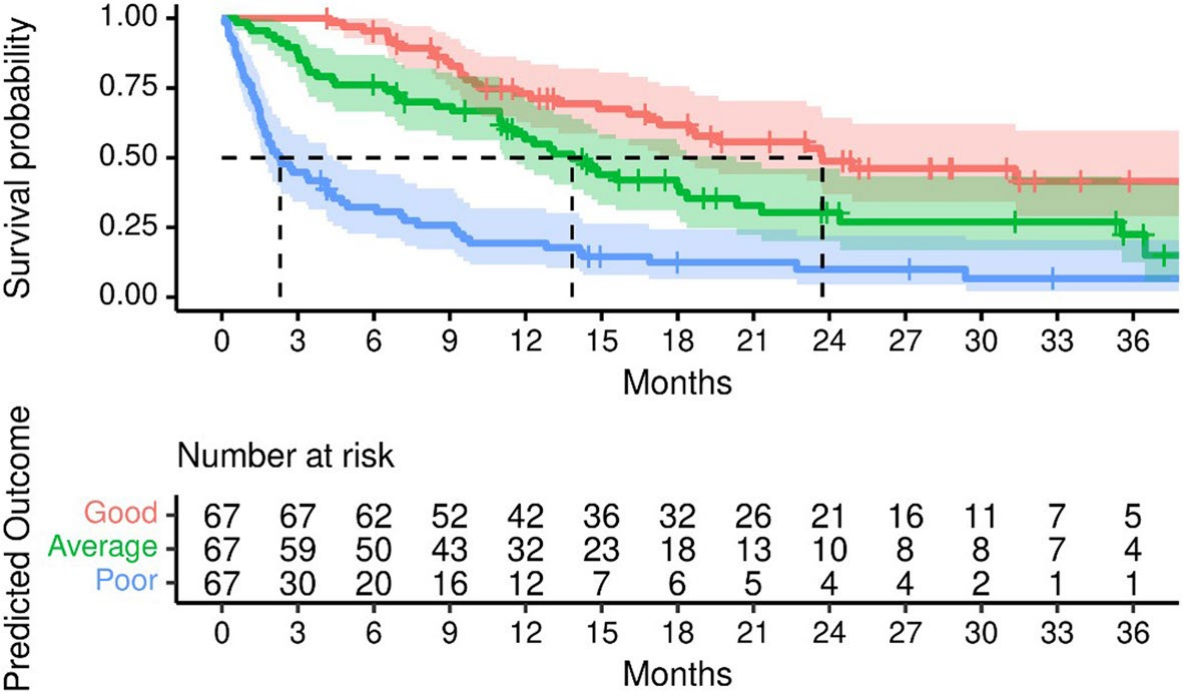
OS in three different risk groups (good, average, poor) stratified at the 33rd and 66th percentiles of model predicted outcomes

Using the prognostic index of the chosen model, we stratified patients into three risk groups at the 0.33 and 0.66 quantiles **(**Fig. 7**).** Median OS in the good risk group was 23.7 months (CI: 18.5, NR), average risk group 13.8 months (CI: 11.1, 20.3) and in the poor risk group 2.3 months (CI: 1.7, 4.4).

## Discussion

Our data expands and temporally validates a previously characterized predictive nomogram for 6 months, 1-year, and 2-years OS in HNSCC patients treated with immunotherapy. We identified several inflammatory clinical and laboratory markers which can predict patient survival. Over the past few years, inflammatory cells have gained significant momentum as major regulators of the TME and immunotherapy response [20]. Immune activation is counterbalanced by factors in the TME that prevent uncontrolled response. Tumors hijack these molecular mechanisms to suppress anti-tumor immunity. In addition to inhibitory checkpoints such as PD-1 and cytotoxic T**-**lymphocyte-associated protein 4 (CTLA 4), key cellular mediators of tumor immune tolerance are myeloid-derived suppressor cells**,** regulatory T-cells, tumor-associated macrophages, and defective antigen-presenting cells [21]. Specifically, these inflammatory cells not only promote direct tumor growth and invasiveness via the enhancement of pro-oncogenic and angiogenic signals [22–24], but they also orchestrate the hallmarks of an immunosuppressive microenvironment. These hallmarks include impairment of T-cell infiltration, activation of an immunosuppressive signaling pathway and enhancement of immunosuppressive metabolism [25].

The neutrophil, as the most prevalent white blood cell, has acquired special attention from investigators and has been studied extensively for years. Historically, its role has been linked to the immune system as a key component of the host defense and the inflammatory cascade. It was not until recently that researchers started to identify its vital role in the TME where neutrophils are playing an essential part. Their contribution to chronic inflammation in the TME leading to cancer progression and its ability to metastasize is well described [26]. Multiple mechanisms were suggested in the neutrophils pro-tumor roles [27]. These mechanisms include neutrophil released enzymes, pro-tumor neutrophil secreted cytokines and the release of reactive oxygen species [27]. Neutrophils being a major player in the TME were also linked to chemotherapy resistance with reports of significant correlation between neutrophilia and poor clinical outcomes [28].

The reduced lymphocyte count in advanced stage malignancies is well established in cancer literature. Multiple studies were able to identify a strong link between lymphopenia and poor outcomes including reduced PFS, OS and strong correlation with poor performance status [29, 30]. In addition; the severity of lymphopenia correlates with worse prognosis and counts as an independent predictor of high toxicity associated with the initiation of chemotherapy [31, 32]. It is strongly believed that cancer cells would thrive in a lymphocyte depleted environment [33, 34]. Several different mechanisms in which the tumor cells may contribute to lymphopenia were described [34]. These mechanisms include direct destruction of lymphocytes by cancer cells through the expression of pro-apoptotic ligands, reduced capacity of lymphocytes to respond to T-cell receptor stimulation, high proportion of CTLA 4 expressing T-regulatory cells leading to immunosuppression and activation induced cell death [34].

Our proposed prediction model demonstrated clear separation of patients into three groups, which underscores the role in the progression and the response to ICI in HNSCC patients for the included predictor variables. Another important point derived from our single institutional cohort is the robust reproducibility and statistical strength. Compared to our original cohort [15], we provide evidence for enhanced goodness of fit in our model. Our cohort is unique since we have collected a series of detailed and inexpensive clinical parameters, widely available and easily applicable in every healthcare setting. More importantly, although nomograms have been developed for the overall survival of HNSCC patients based on gene signatures [35], our cohort is the first to describe a survival nomogram for patients receiving ICI. The utilization of nomograms is an important milestone in the clinical decision-making in patients with malignancies [36], already used in melanoma [37, 38] and lung cancer [39–41]. Given the risk of toxicities described with ICI [42, 43] and their excessive economic burden [44, 45], our nomogram could assist in the precise selection of patients for which ICI could be beneficial, and cost-effective. The importance of our nomogram model could also be expanded in other immunomodulatory therapies that target or modulate the TME. These piloted clinical approaches, which are currently investigated in clinical trials, include T-cell based approaches, immunostimulatory agents, oncolytic viruses, Interleukin agonists, Toll-like Receptor (TLR)/IFN pathway modulators and emerging vaccines.

As mentioned in our preceding sections, our data has some limitations. Our study is a single institutional retrospective analysis, which may be affected by the genetic background of our community and the clinical practices adopted in our center. Although our expanded cohort exhibited expected temporal validation results, further suggesting the importance of our model, independent external validation should occur. There is an emerging role of molecular markers that could act as specific predictors for assessing prognosis and therapeutic response in patients with R/M HNSCC [46, 47]. Our cohort did not include genetic or immunohistochemistry (IHC) studies, such as PD-L1 expression or tumor mutational burden, which we are willing to include in our follow-up studies.

We have temporally validated and expanded our previously described prognostic survival nomogram for HNSCC patients receiving ICI. Our next focus is on an external validation of our model from an independent clinical setting. Furthermore, we are willing to expand our simple, inexpensive and comprehensive group of variables by including a cohort of IHC markers at the time of diagnosis, along with information from the mutational landscape of these patients after Whole Exome Sequencing (WES). Finally, our ultimate goal is to improve the predictive power of our nomogram with the utilization of multi-omics Next Generation Sequencing (NGS) approaches that will identify gene signatures and distinct signaling pathways involved in the regulation of TME and the response of HNSCC patients treated with ICI.

## Data Availability

The datasets generated and/or analysed during the current study are not publicly available due **t**heir containing information that could compromise the privacy of research participants, but are available from the corresponding author on reasonable request.

## Abbreviations

HNSCC: Head and neck squamous cell carcinoma
ICI: Immune checkpoint inhibitors
R/M: Recurrent/metastatic
OS: Overall survival
Hb: Hemoglobin
Alb: Albumin
LDH: Lactate dehydrogenase
TME: Tumor microenvironment
Neu: Absolute neutrophil count
Lymph: Absolute lymphocyte count
PFS: Progression free survival
CTLA 4: Cytotoxic T**-**lymphocyte-associated protein 4
IHC: Immunohistochemistry
